# Long catheter sign: a reliable bedside sign of incorrect positioning of foley catheter in male spinal cord injury patients

**DOI:** 10.1186/1757-1626-1-43

**Published:** 2008-07-17

**Authors:** Subramanian Vaidyanathan, Peter L Hughes, Tun Oo, Bakul M Soni

**Affiliations:** 1 Regional Spinal Injuries Centre, District General Hospital, Southport, PR8 6PN, UK; 2 Department of Radiology, District General Hospital, Southport, PR8 6PN, UK

## Abstract

**Introduction:**

Indwelling urethral catheter is often used in male spinal cord injury patients to provide drainage to neuropathic bladder. If the balloon of a Foley catheter is inflated in urethra or, when a properly inserted Foley catheter is later pulled and thereby, the Foley balloon comes to lie in urethra, an excessive length of catheter will remain outside the penis. This sign is termed "long catheter sign". Long catheter sign will also be positive when Foley catheter slips out of urinary bladder in situations where Foley balloon is ruptured by a spiky vesical calculus or deflated due to a defective valve.

**Case Presentation:**

A fifty-year-old Caucasian male with paraplegia at T-5 level had been managing neuropathic bladder by long-term indwelling urethral catheter. During his stay in spinal unit, the patient felt that there had been a tug on the drainage tube when he was being turned during night as part of the routine care for relief of pressure. Next morning, a health professional noticed that a long segment of catheter was lying outside penis. There was no bleeding from urethral meatus. Catheter continued to drain urine, which was yellowish in colour. Urine output was satisfactory. This patient did not develop any clinical feature of autonomic dysreflexia nor was he feeling unwell. In view of positive long catheter sign, radiological studies were performed to check the position of Foley catheter, which confirmed the clinical impression of incorrectly positioned Foley catheter. The catheter was removed; flexible cystoscopy was performed. A 16 Fr, 20 ml balloon Foley catheter was inserted over a 0.032" guide wire. Following this procedure, a considerably shorter length of Foley catheter remained outside the penis.

**Conclusion:**

Positive long catheter sign indicates that the Foley catheter is placed incorrectly and needs repositioning urgently. Prompt recognition of long catheter sign and immediate repositioning of Foley catheter will help to prevent complications such as chronic distension of urinary bladder, urine infection, and pressure necrosis of urethra especially if Foley balloon remains inflated within urethra for a long period. In this patient, use of a Foley catheter with 20 ml balloon, and securing the drainage tube to thigh with two straps, helped to prevent inadvertent pull of Foley balloon into the urethra.

## Introduction

Management of the neurogenic bladder has the primary objectives of maintaining continence, ensuring low bladder pressure (to avoid renal damage) and avoiding or minimising infection. Options include intermittent urethral catheterisation, indwelling urethral or suprapubic catheterisation, timed voiding, use of external catheter (for men), drug treatment, augmentation cystoplasty and urinary diversion. [1] Long-term indwelling urethral catheterisation is common amongst people with cervical spinal cord injury; however, this carries a high risk of developing a catheter-related urinary tract infection and associated complications especially bypassing and leakage. [2,3]

Carers and health professionals tend to assume that urethral catheter drainage is satisfactory in spinal cord injury patients if the catheter is draining clear urine, there is no blood in urethral meatus, and the patient does not develop features of autonomic dysreflexia. In male spinal cord injury patients, the balloon of a Foley catheter may be inflated in urethra quite inadvertently or, a properly inserted Foley catheter may be pulled and thereby, the Foley balloon may come to lie in urethra. If this happens, an excessive length of Foley catheter will be outside the penis. This sign is termed "long catheter sign". Long catheter sign will also be positive when Foley catheter slips out of urinary bladder in situations where Foley balloon is ruptured by a spiky vesical calculus or deflated due to a defective valve. We present clinical images of long catheter sign in a paraplegic patient in order to raise awareness amongst health professionals of this readily recognisable bedside clinical sign.

## Case Presentation

A fifty-year-old Caucasian male sustained paraplegia at T-5 level in 1987 while driving his car, which skidded and crashed. He had been managing his bladder with long-term indwelling urethral catheter. He developed a false passage in posterior urethra. Therefore, he used undergo flexible cystoscopy and change of catheter over a guide wire. He used to have a size 16 Fr. silicone Foley catheters with 10 ml balloon.

**Figure 1:**
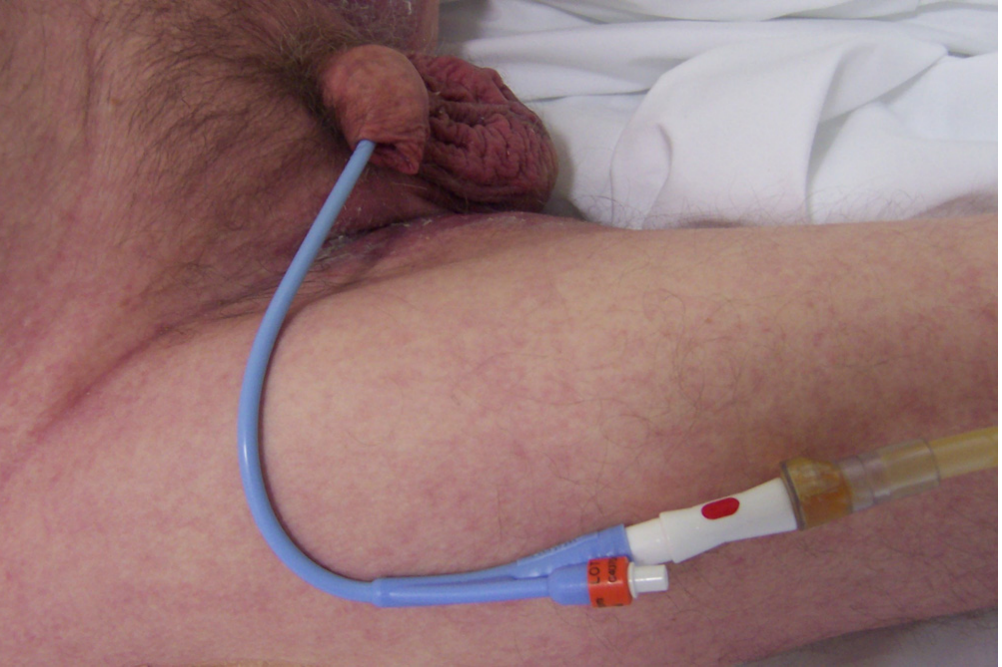
Clinical photograph of genitalia of a spinal cord injury patient showing positive long catheter sign: A very long segment of Foley catheter is lying outside penis.

In 2005, this patient underwent above-knee amputation for pressure sore over left knee joint. He was readmitted to Spinal Injuries Centre in February 2008 for revision of the stump. On one night during his stay in spinal unit, the patient felt that there had been a tug on the drainage tube while he was being turned as part of the routine care for relief of pressure. Next morning, a health professional noticed that a long segment of catheter was lying outside penis. (Figure 1) There was no bleeding from urethral meatus. Catheter continued to drain urine, which was yellowish in colour. Urine output was satisfactory. This patient did not develop any clinical feature of autonomic dysreflexia nor was he feeling unwell.

**Figure 2:**
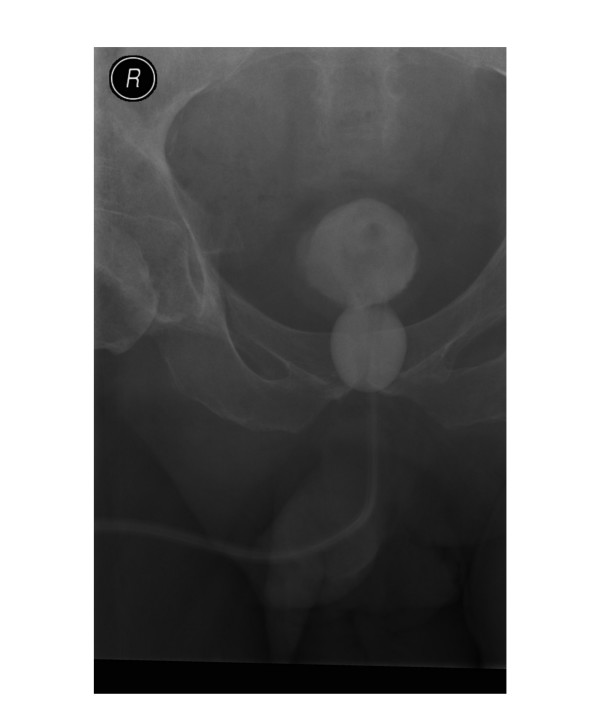
**X-ray of pelvis, taken after injecting two ml of Ioversol (OPTIRAY 300) through balloon channel of Foley catheter and 50 ml of diluted contrast through the main lumen of Foley catheter in order to visualise Foley balloon and urinary bladder respectively**: The outline of urinary bladder is seen clearly. A smaller circular opaque shadow, situated below the urinary bladder, represents the. Foley balloon. The Foley balloon is lying in posterior urethra. When a Foley catheter is positioned correctly, the Foley balloon should lie within the cavity of urinary bladder.

**Figure 3:**
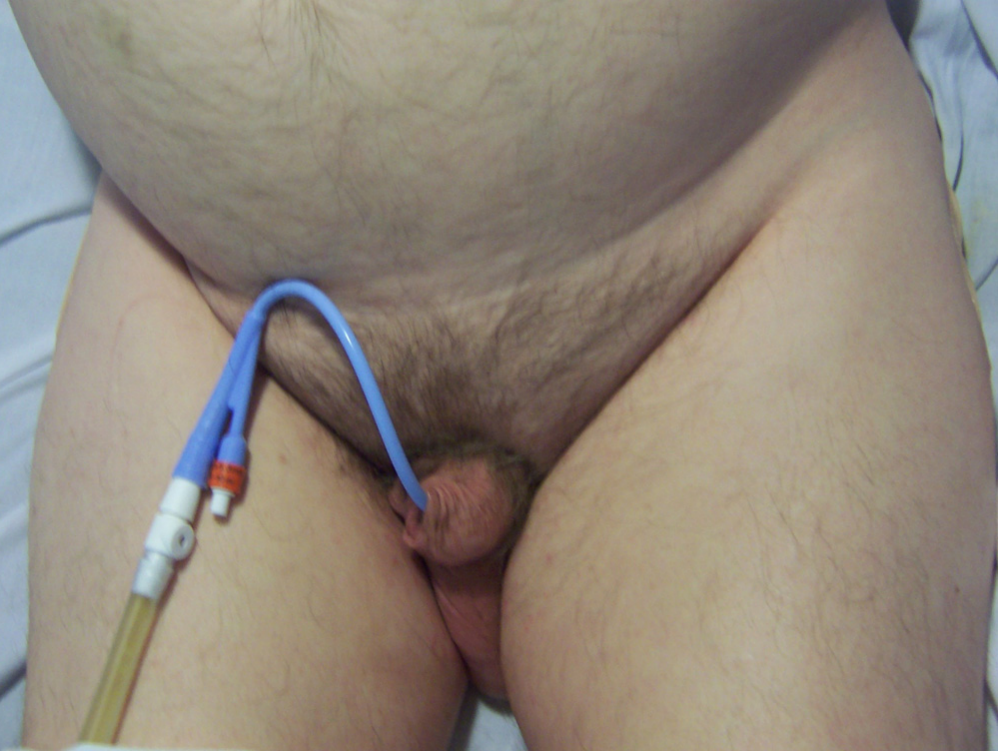
**Clinical photograph of lower abdomen of the same patient as in Figure 1**: The incorrectly placed Foley catheter has been removed. A new Foley catheter has been inserted over a guide wire after performing flexible cystoscopy. When a Foley catheter is correctly placed, considerably shorter segment of the catheter should remain outside penis. This is evident when Figure 1 is compared with Figure 3.

In view of positive long catheter sign, radiological studies were performed to check the position of Foley catheter [4]. X-ray of urinary bladder, taken after injecting contrast both through balloon channel and main lumen of Foley catheter showed the Foley balloon to be lying in urethra. (Figure 2) This radiological study confirmed the clinical impression of incorrectly positioned Foley catheter. The catheter was removed; flexible cystoscopy was performed. A 16 Fr, 20 ml balloon Foley catheter was inserted over a 0.032" guide wire. Following this procedure, a considerably shorter length of Foley catheter remained outside the penis. (Figure 3) Positive long catheter sign could be recognised readily when Figure 1 is compared with Figure 3. Insertion of a Foley catheter with 20 ml balloon, and securing the drainage tube to thigh with two straps, helped to prevent inadvertent pull of Foley balloon into the urethra.

## Discussion

### Clinical relevance of Long Catheter Sign

Carers and health professionals should look for this simple bedside sign while they attend to a male spinal cord injury patient. Positive long catheter sign indicates that the Foley catheter is placed incorrectly and needs repositioning urgently. Prompt recognition of long catheter sign and immediate repositioning of Foley catheter will help to prevent complications such as chronic retention of urine, urine infection, and pressure necrosis of urethra especially if Foley balloon remains inflated within the urethra for a long period.

We hope that these clinical images of long catheter sign in a paraplegic patient will help to raise awareness amongst health professionals of this readily recognisable bedside clinical sign. Further, these clinical images can be used in teaching health professionals, who are involved in the care of spinal cord injury patients in the hospital setting and particularly, in the community.

### Patient's perspective

My problem has been solved after cystoscopy and insertion of a larger balloon catheter.

## Consent

This patient was happy to provide written informed consent for publication of this manuscript and accompanying images in Cases Journal. A copy of the written consent is available for review by the Editor-in-Chief of this journal.

## Competing interests

The authors declare that they have no competing interests.

## Authors' contributions

SV spotted "long catheter sign" and performed radiological study and subsequently, cystoscopy. All authors participated in the care of this patient and contributed to the manuscript.
